# Environmental diagnoses and effective planning of Protected Areas in Brazil: Is there any connection?

**DOI:** 10.1371/journal.pone.0242687

**Published:** 2020-12-11

**Authors:** Ana Rafaela D´Amico, José Eugênio Cortes Figueira, José Flávio Cândido-Jr., Maria Auxiliadora Drumond

**Affiliations:** 1 Coordenação de Elaboração e Revisão de Planos de Manejo, Instituto Chico Mendes de Conservação da Biodiversidade, Brasília, Distrito Federal, Brasil; 2 Laboratório de Sistemas Socioecológicos, Departamento de Genética, Ecologia e Evolução, Instituto de Ciências Biológicas, Universidade Federal de Minas Gerais, Belo Horizonte, Minas Gerais, Brasil; 3 Laboratório de Ecologia de Populações, Departamento de Genética, Ecologia e Evolução, Instituto de Ciências Biológicas, Universidade Federal de Minas Gerais, Belo Horizonte, Minas Gerais, Brasil; 4 Laboratório de Ecologia e Conservação, Departamento de Ciências Biológicas, Universidade Estadual do Oeste do Paraná, Cascavel, Paraná, Brasil; CIRAD, FRANCE

## Abstract

Protected Areas (PAs) are essential to maintaining biodiversity, while effective management plans (MPs) are essential for the management of these areas. Thus, MPs must have relevant data analyses and diagnoses to evaluate ecological conditions of PAs. We evaluated the environmental diagnoses of 126 Brazilian federal PAs, the methods used to collect data and defined the diagnostic level of PMs according to the type and number of analyzes performed for each PA category. We found a low level of diagnosis in MPs. Primary field data or research programs resulted in environmental diagnostics of higher levels. Participatory workshops and secondary data, most used in Extractive Reserves, were related to low levels of diagnoses. The most frequent analysis was the identification of threats (97% of MPs), while the least frequent were the definition of conservation targets and future scenarios for management (1.6% of MPs). Our results show that the diagnoses of the MPs need to be more analytical to generate useful information for decision-making. MPs should prioritize data analysis and specific management studies, focused on the use of natural resources, the status of conservation targets, future scenarios, and key information to planning.

## Introduction

Protected Areas (PAs) are fundamental for conserving biodiversity. If well managed, PAs preserve endangered species, healthy ecosystems and ecological processes, generating benefits including several environmental services [1–5]. However, global conservation efforts face increased human pressures on natural ecosystems continues to decline [6].

A good management plan (MP) is essential for an effective PA and must contain clear strategies to achieve goals for nature conservation and human well-being. Furthermore, MPs should address the context of the region [3,7]. This assessment, or diagnosis, should identify the ecological condition of the PA, values for conservation, social aspects and the threats to the area. It indicates *“where we are”*, and it is key to *“where we want to reach”*, and the *“how we get there”* [8]. Relevant ecological data are needed to support good management [9], but the research produced does not always satisfy the needs of the conservation and decision-makers [10]. However, research on different areas should answer questions for effective management of PAs. These surveys make up the initial phase of planning. Diagnoses can use data from different sources, such as long-term biological inventories, Rapid Ecological Assessments [11], local communities’ knowledge, or workshops with specialists [9,12], produced specifically for the MP (primary data) or from researches or technical reports made for other objectives, does not related to MP (secondary data). However, the information gathered is not always integrated, resulting in only extensive descriptions, which are not very useful for planning the management of a PA. The identification of conservation targets and their threats and the spatial distribution of these elements would be more effective for defining the objectives and results for the conservation of a PA and the management strategies to reach them [12–14]. They should also support the monitoring of conservation effectiveness [8].

The strengthening PA management in Brazil—designated Conservation Units—is particularly important, covering 18.6% of its continental territory and 26.5% of its marine territory [15]. These areas are responsible for conserving megadiversity and ecosystem services of global magnitude [2]. However, the threats to these PAs are increasing, ranging from over-exploitation of biodiversity (hunting, fishing and the collection of timber and non-timber products), habitat loss through the advancement of agribusiness, to large mining and hydroelectric projects [16], and fire, which is increasingly frequent in PAs in fire-sensitive Amazonian forests [17], or in fire-prone ecosystems, like the Brazilian Cerrado [18].

About half of the 334 PAs managed by the Brazilian federal government have MPs. A main barrier to the MPs has been elaboration of diagnoses of the physical, biological and socioeconomic aspects, since they require a long time, financial resources and have been of little use for effective PA planning. However, despite criticism of this important planning stage, there are no deeper assessments of its design and there are no concrete proposals to change it. In this work, we present an overview of the environmental diagnosis carried out in support of MPs by evaluating the methods used and the analyses carried out to support management decisions. We also make recommendations for improving the process of developing MPs, to contribute to more effective nature conservation.

## Material and methods

### Review of management plans

**Fig 1:**
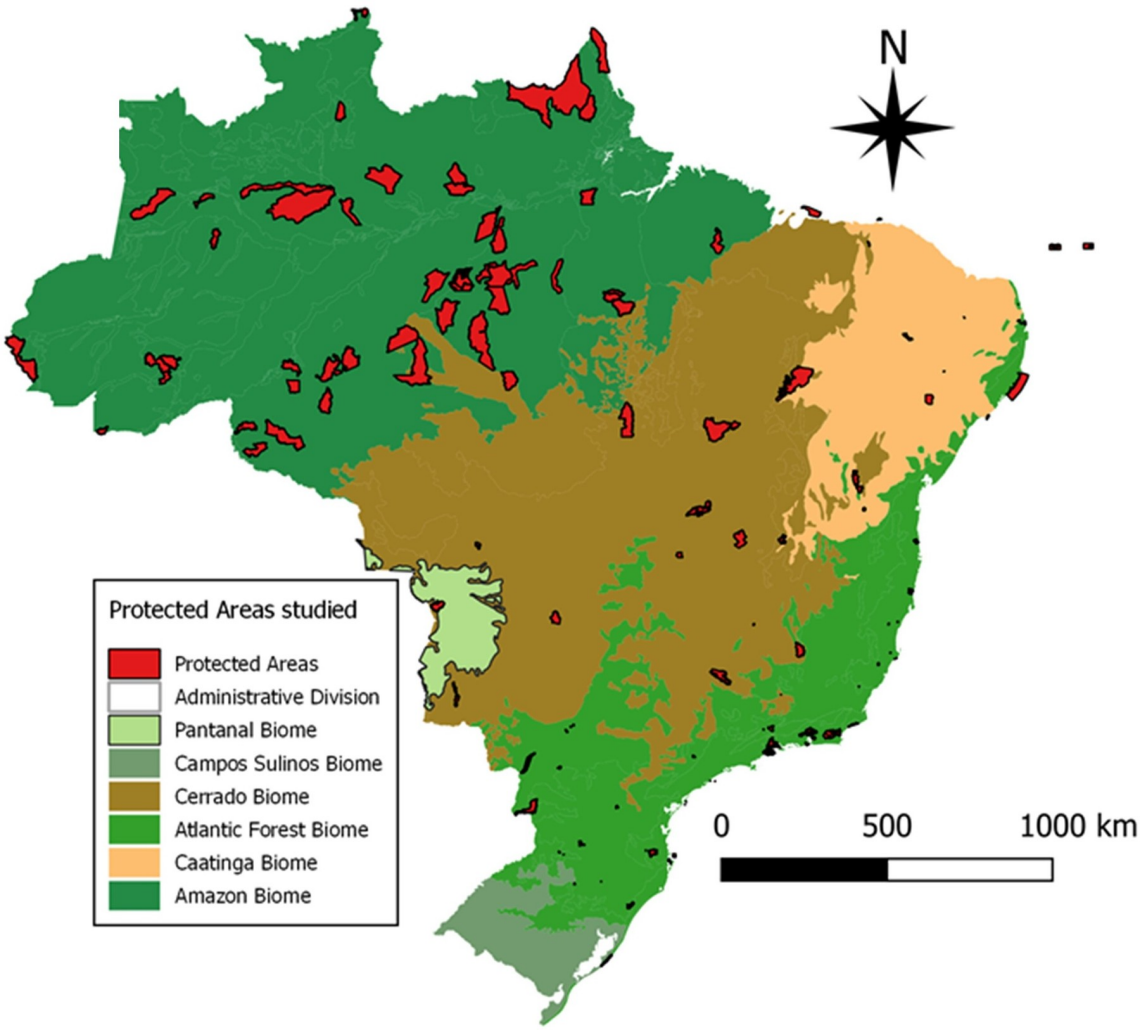
Brazilian biomes and location of the 126 Protected Areas (PAs) that had their management plans evaluated in the present study.

We analyzed 126 MPs of Brazilian PAs (Fig 1), considering only plans approved after the establishment of the National System of Nature Conservation Units, in the year 2000, and until December 2014. We obtained the MPs from the ICMBio’s website (*www.icmbio.gov.br*). Then, we identified the procedures used in the environmental diagnoses in the MPs or in the administrative processes made available by ICMBio, the methods used for data collection (primary and/or secondary) and the analyses performed. The 126 Brazilian federal Protected Areas administered by ICMBio whose management plans were analyzed in this study represents 38% of these areas and were classified according to Classes, Brazilian categories and IUCN categories [19]. Therefore, Full Protection PAs encompassed 20 Biological Reserves (6% of Brazilian Federal PAs) and 12 Ecological Stations (4% of Brazilian Federal PAs), corresponding to Ia-IUCN, and 39 National Parks (12% of Brazilian Federal PAs)—II-IUCN. On the other hand, Sustainable Use PAs encompassed 11 Environmental Protection Areas (3% of Brazilian Federal PAs)—V-IUCN, 28 National Forests (8% of Brazilian Federal PAs) and 16 Extractive Reserves (5% of Brazilian Federal PAs)- VI-IUCN.

### Methods used in diagnoses

The methods used in the environmental diagnoses were classified into seven categories: (1) participatory workshops or interviews with residents or beneficiaries of the PA; (2) use of secondary data on the region of the PA; (3) use of a few secondary data from the interior of the PA; (4) specific studies for management of species, biological communities or natural resources; or use of information from previous studies; (5) primary data from rapid surveys; (6) primary data from long-term surveys; and (7) use of secondary data from PA research programs, which consist of extensive research, in which a large amount of information is accumulated—this differs this category from those (2) and (3) (S2).

### Diagnostic analysis

We identified five types of analyses recommended in the MP environmental diagnoses: 1) identification of biodiversity threats [8,13]; 2) definition of conservation targets [8,13]; 3) classification of PA environments, that may occur from three different forms: according to biological importance, state of conservation or vulnerability [11,20]; 4) future management and conservation scenarios [8,20]; and 5) integrated analysis of the different diagnostic themes [11] S3. Each one of these analyses received a 0 or 1 score, and a PA has a better diagnosis level when several of these analyses were performed. The level of diagnosis may vary from 0 to 5, obtaining the maximum value when all analyzes are done.

Once to know and combat threats is the essence of any conservation project, especially in PAs, where they hamper the maintenance of their value and where it is necessary to monitor them to evaluate the conservation effectiveness of the area [1,21], we scored as 0 the PAs with “no threats identified”.

### Data analysis

We used the G-test to evaluate the relationship between the categories of PAs and the methods and analysis of diagnoses, and compared the variation in the level of diagnosis among the categories of PAs by the Kruskal-Wallis test, using the program BioEstat 5.0 [22].

A One-way Analysis of Similarity—ANOSIM [23] with sequential Bonferroni correction was used to test for differences among PAs based on the combination of types of analysis and methods of diagnoses, using the software PAST [24].

Non-metric multidimensional scaling (NMDS) was used to analyze the relationship between PA categories, methods and diagnoses, performed in PC-Ord 5.10 in the “slow and through” mode, using Jaccard distances. The number of dimensions were defined by the final stress values that were compared with randomized runs of the data set [25], and also with stress values considered significant and satisfactory [26,27]. Pearson’s correlations of each variable with ordination axes were used to axes interpretation. Here, we excluded the method that was used in all MPs and primary long-term studies that were used in just two MPs.

## Results

### Diagnosis data

Secondary data on the region of the PA was used in the development of all of the MPs, with the majority of them (79%) also using a few secondary data on the PA itself. To complement this information, was used primary data from rapid surveys (67%) and specific management studies (32%). Participatory workshops with beneficiaries and secondary data from research programs were also little used with 15% and 10%, respectively. With the exception of one MP, the diagnoses employed two or more methods (Table 1).

**Table 1:** Methods of obtaining data in diagnoses by each protected area category. Figures are the number of times each method was used and their respective percentages (in parentheses).

*Category*	Number of PAs in each category	Part-Work	Sec-Data-Reg	Sec-Data	Man-Stud	Rap-Surv	Long-Surv	Sec-Res
Environmental Protection Areas	11	3 (27)	11 (100)	8 (73)	4 (36)	4 (36)	0 (0)	2 (18)
Ecological Stations	12	0 (0)	12 (100)	10 (83)	2 (17)	8 (67)	0 (0)	1 (8)
National Forests	28	1 (4)	28 (100)	18 (64)	19 (68)	23 (82)	0 (0)	5 (18)
National Parks	39	0 (0)	39 (100)	31 (79)	6 (15)	33 (85)	2 (5)	4 (10)
Biological Reserves	20	0 (0)	20 (100)	19 (95)	2 (10)	12 (60)	0 (0)	1 (5)
Extractive Reserves	16	15 (94)	16 (100)	13 (81)	7 (44)	4 (25)	0 (0)	0 (0)
Total	126	19 (15)	126 (100)	99 (79)	40 (32)	84 (67)	2 (2)	13 (10)

Part-Work–participatory workshops or interviews with residents or beneficiaries; Sec-Data-Reg–secondary data on the region of the PA; Sec-Data–secondary data on the interior of the PA (few information); Man-Stud–specific management studies; Rap-Surv–primary data from rapid surveys for the MP; Long-Surv—primary data from long-term surveys; Sec-Res -secondary data from PA research programs.

Methods for obtaining data were significantly related to PA category (G-test = 93.20; GL = 30; p < 0.0001). Participatory workshops or interviews and secondary data were more used in Extractive Reserves. Primary data from rapid surveys was most widely used in National Parks, National Forests, Biological Reserves and Ecological Stations. Specific management studies were most commonly used in National Forests and secondary data from research programs was most widely used in National Parks and National Forests. Environmental Protection Areas were not related to a specific method (Table 1).

### Diagnostic analyses

The most frequent analyses were the identification of threats (97.6%) and the classification of environments (73%), in general, by their state of conservation (69%). Integrated analysis of themes (19%), identification of conservation targets (1.6%) and the evaluation of future scenarios (1.6%) were less used. In only two PAs no analyses were performed (Table 2). There was no relationship between the types of analysis and PA category (G-test = 31.73; GL = 30; p = 0.37).

**Table 2:** Number and percentage (in parentheses) of analyses performed in environmental diagnoses of management plans by category of Protected Areas.

*Category*	*Diagnostic analyses*						
	*Threats*	*Conservation targets*	*Environmental classification*			*Future scenarios*	*Integrated analysis of themes*
			*Biological importance*	*Vulnerability*	*State of conservation*		
Environmental Protection Areas	11 (100)	0 (0)	1 (9.1)	5 (45.5)	8 (72.7)	0 (0)	0 (0)
Ecological Stations	12 (100)	0 (0)	2 (16.7)	3 (25.0)	8 (66.7)	0 (0)	1 (8.3)
National Forests	27 (96.4)	0 (0)	4 (14.3)	9 (32.1)	24 (85.7)	1 (3.6)	8 (28.6)
National Parks	39 (100)	1 (2.6)	12 (30.8)	14 (35.9)	24 (61.5)	1 (2.6)	12 (30.8)
Biological Reserves	20 (100)	1 (5.0)	3 (15.0)	4 (20.0)	13 (65.0)	0 (0)	3 (15.0)
Extractive Reserves	14 (87.5)	0 (0)	0 (0)	1 (6.3)	10 (62.5)	0 (0)	0 (0)
Total	123 (97.6)	2 (1.6)	22 (14.5)	36 (28.6)	87 (69.0)	2 (1.6)	24 (19.0)

### Level of diagnosis

**Fig 2:**
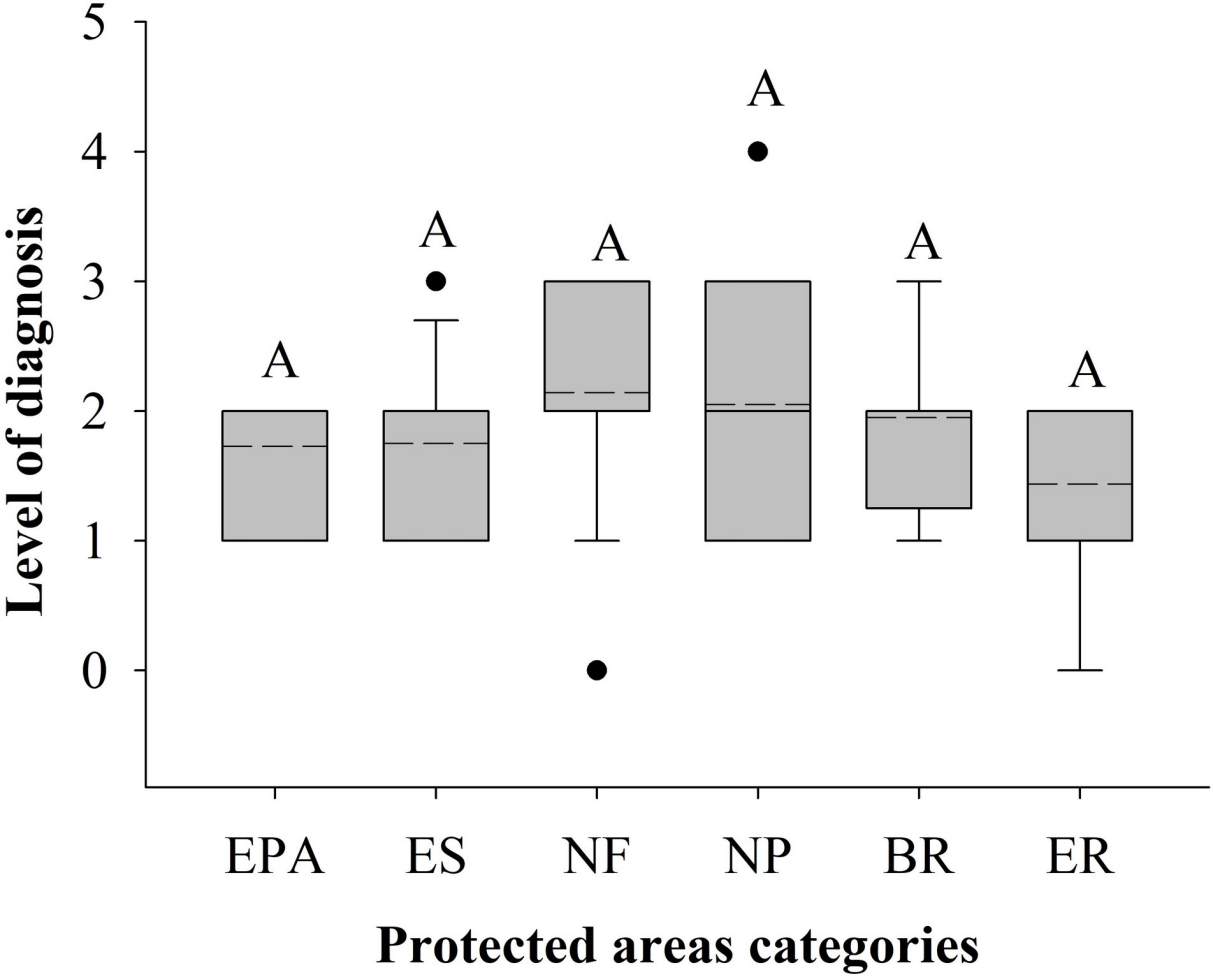
Level of diagnosis of categories of protected areas. Dashed lines represent means. A = values that do not differ statistically (H = 10.51; GL = 5; p = 0.061). Categories: EPA–Environmental Protection Areas, ES–Ecological Stations, NF–National Forests, NP–National Parks, BR–Biological Reserves, ER–Extractive Reserves.

In only three MPs (2.4%) no biodiversity threats were identified (diagnosis level 0), while in 25.4% of the MPs only this analysis was done (level 1). Threat identification and environment classification were performed in 50.8% of the MPs (level 2). We recorded threat identification, environment classification, and a third analysis, mainly the integration of themes in 20.6% of the MPs (level 3). Only one MP (0.8%) had four types of analyses (level 4), and none had five. The average level of diagnosis for the MPs was 2, with no differences in means among PA categories (H = 10.51; GL = 5; p = 0.061) (Fig 2).

### Relationships among categories of PAs and methods of diagnosis

There were significant differences among PAs according to analysis and methods of diagnoses: ANOSIM’s R = 0.199, p-value = 0.001, and Table 3.

**Table 3:** P-values of ANOSIM-pairwise comparisons with Bonferroni correction between Protected Areas categories, according to analysis and methods of diagnoses.

	EPA	ES	NF	NP	BR	ER
EPA						
ES	1,0000					
NF	0,1380	1,0000				
NP	0,0975	1,0000	**0,0465**			
BR	0,5970	1,0000	**0,0405**	1,0000		
ER	0,1170	**0,0015**	**0,0015**	**0,0015**	**0,0015**	

Significant differences are highlighted in bold. Categories: EPA–Environmental Protection Areas, ES–Ecological Stations, NF–National Forests, NP–National Parks, BR–Biological Reserves, ER–Extractive Reserves.

**Fig 3:**
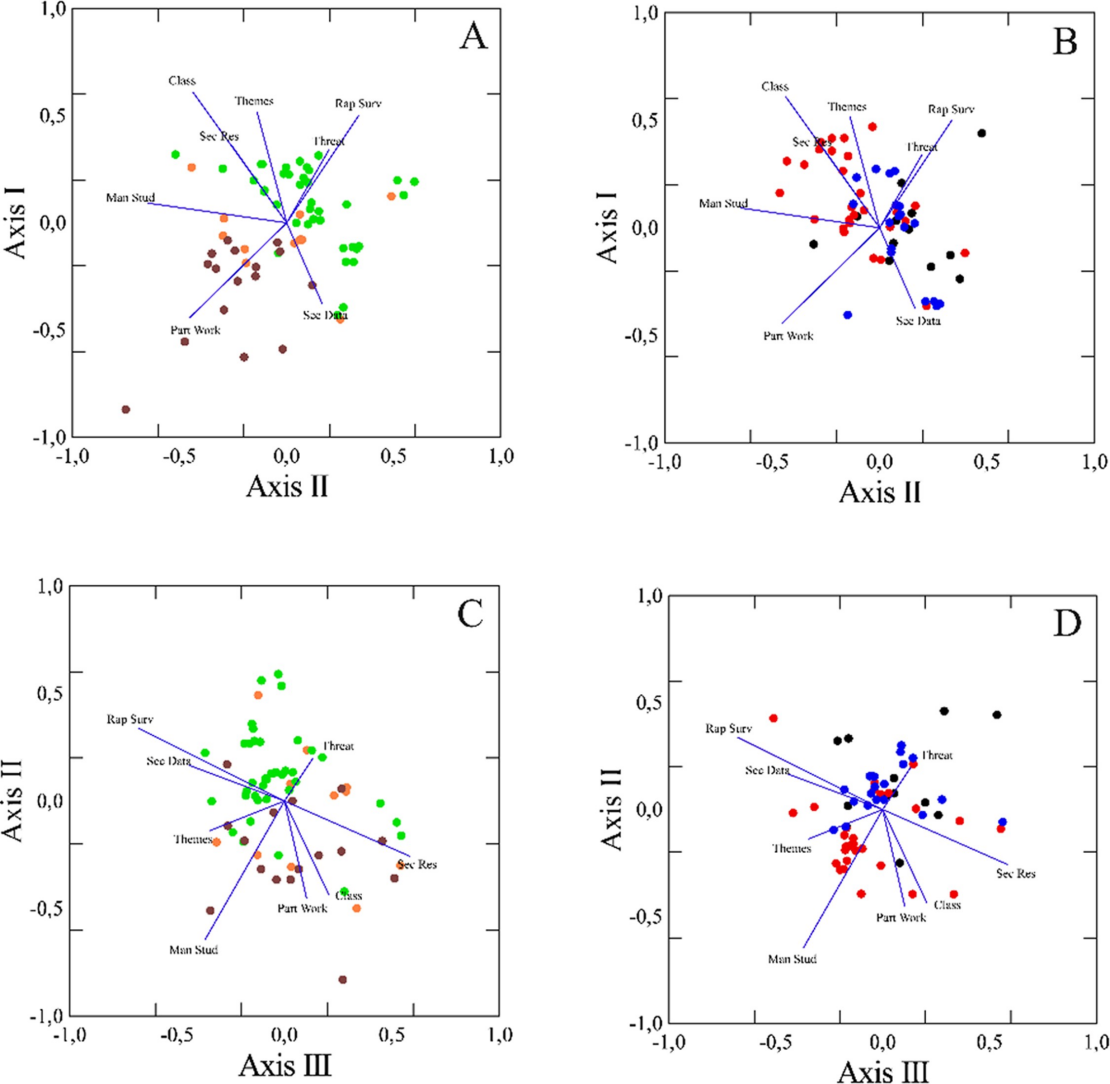
Ordination of Protected Areas (PAs) relative to NMDS Axis I versus II (A and B), and Axis II versus III (C and D). The colors represent PA categories: orange–Environmental Protection Areas; black–Ecological Stations; red–National Forests; green–National Parks; blue–Biological Reserves; brown–Extractive Reserves. Methods: Part-Work–participatory workshops or interviews with residents or beneficiaries; Sec-Data–secondary data on the interior of the PA (little information); Man-Stud–specific management studies; Rap-Surv–primary data from rapid surveys for the MP; Sec-Res–secondary data from PA research programs. Threat—identification of biodiversity threats; Class—classification of PA environments; Themes—integrated analysis of the different diagnostic themes. Axes are scaled according to Pearson's correlation coefficients (vectors) between each method and the Axis. Due to a large number of overlapping, data points were 'jittered' on both axes, by adding random noise to their coordinates.

The NMDS algorithm recommended a three-dimensional solution with final stress = 11.958, p-value = 0.040. For the sake of clarity, we present pairs of axes (Axis I versus II, and Axis II versus III, each one split into three categories of PAs), of the NMDS three-dimensional solution (Fig 3). These axes distinguished the categories of PAs in loose, but relatively coherent groups according to methods and level of diagnosis (Table 4). We can notice a contrast between National Forests, National Parks and Biological Reserves characterized by the use of primary data from Rapid Surveys and high diagnosis level (threat identification, environment classification and mainly integrated analyses of themes), and Extractive Reserves. In addition, Management Studies, Participatory Workshops and the variable class of diagnosis level were more usual in Environmental Protection Areas, National Forests and Extractive Reserves. There was a tendency for a higher level of diagnosis in MPs that used primary data from rapid surveys or secondary data from PA research programs. Plans that adopted participatory workshops, specific management studies, and few secondary data were related to lower levels of diagnosis. Corroborating the ANOSIM results, the points which correspond to Extractive Reserves on the NMDS three-dimensional plot appear grouped differently from other PA categories, except for Environmental Protected Areas whose points do not display a clear pattern.

**Table 4:** Pearson’s correlations of each variable and axes I, II and III of the NMDS analysis.

Squared correlations between ordination distances and distances in three-dimensional space:			
	0,394	0,288	0,206
Variable	Correlations with ordination axes:		
	Axis I	Axis II	Axis III
Part-Work	**-0,442**	**-0,452**	**0,103**
Sec-Data	-0,375	**0,165**	**-0,442**
Man-Stud	**0,092**	**-0,646**	**-0,370**
Rap-Surv	**0,501**	0,337	**-0,678**
Sec-Res	**0,362**	-0,257	**0,584**
Threat	**0,341**	**0,198**	0,132
Class	**0,609**	**-0,437**	0,207
Themes	**0,517**	-0,138	-0,347

Methods: Part-Work–participatory workshops or interviews with residents or beneficiaries; Sec-Data–secondary data on the interior of the PA (little information); Man-Stud–specific management studies; Rap-Surv–primary data from rapid surveys for the MP; Sec-Res–secondary data from PA research programs; Threat–threat identification; Class–environment classification and Themes–mainly integrated analyses of themes. Correlations in bold were used to interpret the axes.

## Discussion

The use of secondary data in most diagnoses shows there is some scientific knowledge about Brazilian PAs. Especially in Extractive Reserves, the basic information for planning was also derived from participatory workshops. Local knowledge is an important source of information about PAs, but it must be combined with scientific evidence in planning [9,28]. Information such as the amount extracted from the resource and maximum sustainable yield are needed for sustainable management and to reach objectives of biodiversity conservation [29]. However, most Extractive Reserves lack specific management studies and the few existing cover few exploited resources. Although these communities use natural resources over many years, keeping the area apparently preserved, we can not assume that its use poses no risk. Instead, the use of resources, especially when linked to a commercial chain, can become a complex long-term problem depending on the scale, type of use, and the fragility of the environments and species used [30]. All uses of biodiversity, legal or illegal, if not monitored, may affect the ability of a PA to achieve its conservation objectives [7]. Furthermore, the greater the risk or more serious consequences of management actions, the greater the need for scientific data and analyses to support decision-making [31]. It is necessary to equalize the objectives of Extractive Reserves to ensure the achievement of the Goals of Aichi but also seeking results for conservation of nature beyond social results [29,30].

The rarity of MPs using secondary data from research programs to support management, monitoring and conservation of these areas demonstrates a weakness in the Brazilian PA system [9]. Since extensive research is a barrier to prepare MPs due to the time required for data collection and analysis, the generation of scientific knowledge should be a constant and priority action of management programs. The absence of these programs makes it difficult to monitor conservation effectiveness and is a gap for adaptive management. In addition, access to scientific information for decision-making and knowledge transfer among research institutions, conservation planners and managers should be improved [32].

The use of the data is even more important than the methods. Despite the environmental characterization presented for all PAs, the level of diagnosis is low, independent of PA category. The description of PAs is prioritized to the detriment of data analysis and generate little information for decision-making. The amount of data presented in MPs does not necessarily mean there is quality management information, since the information may not be relevant to the management challenges of the PA. The insufficiency of analysis in environmental diagnoses shows that, even when descriptions of the ecological elements of PAs are made, they are not very effective in the analysis of this information and its transformation into useful information for planning. In many cases, the data obtained are general and purely descriptive, not providing the necessary information for decision making by managers. It would be relevant information, for example, analysis of threats on species or environments and their consequences and possible strategies to minimize them. Data obtained in a targeted and well-analyzes manner are essential to support decision-making for the management of natural areas.

Identification of threats provides key information, but although it was widely used among MPs, it has been presented in most of the plans in a scattered manner, non-systematized, without clear connection between threats and their impacts on conservation. Moreover, in general threats were not related to their real causes, leading planners to focus on the problem rather than on its origin. Unfortunately, the roots of threats also tend to be ignored when they do not fit in the governance of the planning team or PA [5].

Most of the PAs, especially National Parks, Biological Reserves, Ecological Stations and National Forests, provide statements of significance in their diagnoses, but these are very comprehensive and are more of a description of the PA than an indication of special elements designated as conservation targets. In these same categories, specific management objectives are usually identified, in some cases together with the researchers who worked on the diagnoses. However, these goals tend to be general and encompass all threatened or special species in the area. Conservation targets and their threats, unusual in Brazilian management plans, should be the basis for the definition of the objectives and results for management of a PA [12–14]. In some countries, such as Costa Rica, Chile, and Argentina, the definition of conservation targets is the first step in the diagnosis. At that moment the information gaps guide the analyses to be carried out are identified [14,33,34].

Classification of PA environments should complement the identification of targets and threats and as a basis for zoning. Among the three possible classifications, state of conservation predominated, likely because it is easier. In general, this is done by analyzing land use and occupation based on vegetation maps, satellite images and deforestation data. Although timber-based assessments allow the identification of areas with different vegetative stages, they do not consider the ecological integrity of environments, which is important for conservation monitoring of PAs [1,5]. Most classifications by biological importance were done using Rapid Ecological Assessments, where researchers regarded the presence of endangered and endemic species. Vulnerability analyses did not show a pattern and were done according to soil fragility and erosion, and with other elements of geology and geomorphology.

One should not neglect future risks and opportunities in diagnoses, especially with climate change, pressure from large enterprises and the advancing deforestation in Brazil [2,16,35], and actions needed to avoid or enhance them [8].

The evaluation of the themes in the diagnoses should be done in an integrated way, to define conservation targets appropriate to the planning. Although there is a trend towards a higher level of diagnosis with the use of methods with primary data collection and long-term research programs, the analyses should be widely performed regardless of the method and amount of information available. Due to scarce resources for conservation, the high cost of data collection in the field and the need for faster and strategic planning in the face of increasing pressures on biodiversity [36]. To improve diagnoses, we recommend integrated analyses with socioeconomic aspects. As ecological aspects are directly influenced by the social context, effective management decisions must consider the socioeconomic dynamics of the PA. Especially in developing countries, where essential management actions, such as land acquisition and protection, are hampered by the scarcity of resources, it is essential to plan based on existing environmental and social information, allocating the few resources available to invest in the analysis of information and adaptive management of PA. Diagnostic analyzes need to be carried out in a better way to achieve a balance between more efficient PMs, which serve as a guide for managers and which are less costly in time and financial resources.

It becomes essential to protect and manage what is not known sufficiently [11], by performing diagnoses and planning of PAs with the best available information, even if it is just secondary data. For this, adaptive management is essential because, as new information and learning are generated, the planning of PAs should be reviewed and improved [8]. One caution about using the best information available to support management is that more conservative measures regarding the use of biodiversity should be prioritized until necessary information is obtained for the safe management of resources. In reserves with communities that depend on resources, investments in research and monitoring should be prioritized. With the recent evidence that assessments of management effectiveness in Brazil do not necessarily reflect conservation results [37,38], the need to improve environmental diagnoses, specially analyses, for planning PAs is even greater. Thus, not only assessments of the effectiveness of PAs for conservation will be facilitated [39], but there will also be gains for biodiversity conservation.

## Conclusions

To better connect the diagnoses and the effectiveness of the PAs, MP development should consider: how threats affect species and ecosystems, how these elements are distributed across the territory of PAs and why some actions are needed to mitigate them; use of methods that facilitate the process of analysis, with analysis of causes and consequences of different factors on conservation targets; classification of PA environments should complement previous the analyses above mentioned and provide the basis for zoning; future scenarios should not be neglected in diagnoses considering climate change, development projects and economic and political cycles; different diagnostic topics should be analyzed in an integrated way; the concepts and practices of adaptive management should be the basis of the planning process, including in the diagnostic phase. The absence of these elements leads to the prioritization of MP activities according to the intuition or experience of managers, with ease of execution, or even disregarding indicated recommendations, impairing management.

To avoid the general problem described here, MPs should prioritize data analysis and use of specific management studies. These studies must focus on the use of natural resources, the status of conservation targets, future scenarios and key information to planning. The same diagnostic and planning methods should be used for all categories of PAs in order to reduce discrepancies between levels of diagnosis and planning and, thus, contribute to the management effectiveness of National Protected Areas Systems.

## Supporting information

S1 Table Protected Areas that had their management plans evaluated in the present study. (DOCX)

S2 Table Criteria for classifying of data collection methods in the environmental diagnoses of management plans. (DOCX)

S3 Table Criteria for classification of the analyses performed in the environmental diagnoses of the evaluated management plans. (DOCX)
