# Supplementary material for: Environmental diagnoses and effective planning of Protected Areas in Brazil: Is there any connection?
Source: PLoS One. 2020 Dec 11;15(12):e0242687. doi: 10.1371/journal.pone.0242687 (PMC7732074; doi:10.1371/journal.pone.0242687)
Supplement: S1 Table — (DOCX) [file pone.0242687.s001.docx]

**S1 Table**. Protected Areas (PA) that had their management plans evaluated in the present study. ^1^ IUCN [36], ^2^ National System of Conservation Units (SNUC).

| IUCN¹  category | SNUC² category and name of protected area | Biome | Year of approval of management plan |
| --- | --- | --- | --- |
| Ia | **Biological Reserves**  Reserva Biológica Atol das Rocas | Marine-Coastal | 2009 |
| Ia | Reserva Biológica Augusto Ruschi | Atlantic Forest | 2002 |
| Ia | Reserva Biológica das Perobas | Atlantic Forest | 2012 |
| Ia | Reserva Biológica de Comboios | Marine-Coastal | 2002 |
| Ia | Reserva Biológica de Poço das Antas | Atlantic Forest | 2005 |
| Ia | Reserva Biológica de Saltinho | Atlantic Forest | 2003 |
| Ia | Reserva Biológica de Serra Negra | Caatinga | 2011 |
| Ia | Reserva Biológica de Una | Atlantic Forest | 2002 |
| Ia | Reserva Biológica do Córrego do Veado | Atlantic Forest | 2000 |
| Ia | Reserva Biológica do Córrego Grande | Atlantic Forest | 2000 |
| Ia | Reserva Biológica do Gurupi | Amazon | 2002 |
| Ia | Reserva Biológica do Jaru | Amazon | 2010 |
| Ia | Reserva Biológica do Rio Trombetas | Amazon | 2004 |
| Ia | Reserva Biológica do Tapirapé | Amazon | 2010 |
| Ia | Reserva Biológica do Tinguá | Atlantic Forest | 2006 |
| Ia | Reserva Biológica do Uatumã | Amazon | 2002 |
| Ia | Reserva Biológica Guaribas | Atlantic Forest | 2003 |
| Ia | Reserva Biológica Marinha do Arvoredo | Marine-Coastal | 2004 |
| Ia | Reserva Biológica Nascentes Serra do Cachimbo | Amazon | 2009 |
| Ia | Reserva Biológica União | Atlantic Forest | 2008 |
| Ia | **Ecological Stations**  Estação Ecológica da Guanabara | Marine-Coastal | 2012 |
| Ia | Estação Ecológica de Aracuri-Esmeralda | Atlantic Forest | 2008 |
| Ia | Estação Ecológica de Carijós | Marine-Coastal | 2003 |
| Ia | Estação Ecológica de Pirapitinga | Cerrado | 2013 |
| Ia | Estação Ecológica de Tamoios | Marine-Coastal | 2006 |
| Ia | Estação Ecológica do Seridó | Caatinga | 2005 |
| Ia | Estação Ecológica dos Tupiniquins | Marine-Coastal | 2010 |
| Ia | Estação Ecológica Juami-Japurá | Amazon | 2002 |
| Ia | Estação Ecológica Mico Leão Preto | Atlantic Forest | 2008 |
| Ia | Estação Ecológica Raso da Catarina | Caatinga | 2008 |
| Ia | Estação Ecológica Rio Acre | Amazon | 2010 |
| Ia | Estação Ecológica Serra Geral do Tocantins | Cerrado | 2014 |
| II | **National Parks**  Parque Nacional Cavernas do Peruaçu | Caatinga | 2005 |
| II | Parque Nacional da Chapada Diamantina | Caatinga | 2009 |
| II | Parque Nacional da Chapada dos Guimarães | Cerrado | 2009 |
| II | Parque Nacional da Chapada dos Veadeiros | Cerrado | 2009 |
| II | Parque Nacional da Lagoa do Peixe | Marine-Coastal | 2004 |
| II | Parque Nacional da Serra da Bocaina | Atlantic Forest | 2002 |
| II | Parque Nacional da Serra da Bodoquena | Cerrado | 2013 |
| II | Parque Nacional da Serra da Canastra | Cerrado | 2005 |
| II | Parque Nacional da Serra da Cutia | Amazon | 2008 |
| II | Parque Nacional da Serra do Cipó | Cerrado | 2009 |
| II | Parque Nacional da Serra do Divisor | Amazon | 2002 |
| II | Parque Nacional da Serra do Itajaí | Atlantic Forest | 2009 |
| II | Parque Nacional da Serra dos Órgãos | Atlantic Forest | 2008 |
| II | Parque Nacional da Serra Geral | Atlantic Forest | 2004 |
| II | Parque Nacional da Tijuca | Atlantic Forest | 2008 |
| II | Parque Nacional das Araucárias | Atlantic Forest | 2010 |
| II | Parque Nacional das Emas | Cerrado | 2005 |
| II | Parque Nacional de Anavilhanas | Amazon | 2002 |
| II | Parque Nacional de Aparados da Serra | Atlantic Forest | 2004 |
| II | Parque Nacional de Ilha Grande | Atlantic Forest | 2008 |
| II | Parque Nacional de Jericoacoara | Marine-Coastal | 2011 |
| II | Parque Nacional de Pacaás Novos | Amazon | 2009 |
| II | Parque Nacional de Ubajara | Caatinga | 2002 |
| II | Parque Nacional Descobrimento | Atlantic Forest | 2014 |
| II | Parque Nacional do Araguaia | Cerrado | 2004 |
| II | Parque Nacional do Cabo Orange | Marine-Coastal | 2011 |
| II | Parque Nacional do Iguaçu | Atlantic Forest | 2002 |
| II | Parque Nacional do Jaú | Amazon | 2002 |
| II | Parque Nacional do Juruena | Amazon | 2011 |
| II | Parque Nacional do Monte Roraima | Amazon | 2000 |
| II | Parque Nacional do Pantanal Matogrossense | Pantanal | 2004 |
| II | Parque Nacional dos Campos Amazônicos | Amazon | 2011 |
| II | Parque Nacional dos Lençóis Maranhenses | Marine-Coastal | 2003 |
| II | Parque Nacional Grande Sertão Veredas | Cerrado | 2003 |
| II | Parque Nacional Itatiaia | Atlantic Forest | 2014 |
| II | Parque Nacional Montanhas do Tumucumaque | Amazon | 2010 |
| II | Parque Nacional Restinga de Jurubatiba | Marine-Coastal | 2008 |
| II | Parque Nacional Serra das Confusões | Caatinga | 2004 |
| II | Parque Nacional Viruá | Amazon | 2014 |
| V | **Environmental Protection Areas**  Área de Proteção Ambiental Anhatomirim | Marine-Coastal | 2013 |
| V | Área de Proteção Ambiental Barra do Rio Mamanguape | Marine-Coastal | 2014 |
| V | Área de Proteção Ambiental Costa dos Corais | Marine-Coastal | 2013 |
| V | Área de Proteção Ambiental da Bacia do Rio Descoberto | Cerrado | 2014 |
| V | Área de Proteção Ambiental da Bacia do Rio São João - Mico Leão | Atlantic Forest | 2008 |
| V | Área de Proteção Ambiental de Cairuçu | Marine-Coastal | 2005 |
| V | Área de Proteção Ambiental de Fernando de Noronha | Marine-Coastal | 2005 |
| V | Área de Proteção Ambiental de Guapi-Mirim | Marine-Coastal | 2004 |
| V | Área de Proteção Ambiental de Petrópolis | Atlantic Forest | 2007 |
| V | Área de Proteção Ambiental de Piaçabuçu | Marine-Coastal | 2010 |
| V | Área de Proteção Ambiental Morro da Pedreira | Cerrado | 2014 |
| VI | **National Forests**  Floresta Nacional Altamira | Amazon | 2012 |
| VI | Floresta Nacional de Amapá | Amazon | 2014 |
| VI | Floresta Nacional de Carajás | Amazon | 2004 |
| VI | Floresta Nacional de Chapecó | Atlantic Forest | 2013 |
| VI | Floresta Nacional de Caxiuanã | Amazon | 2013 |
| VI | Floresta Nacional de Contendas do Sincorá | Caatinga | 2006 |
| VI | Floresta Nacional de Goytacazes | Atlantic Forest | 2013 |
| VI | Floresta Nacional de Ibirama | Atlantic Forest | 2009 |
| VI | Floresta Nacional de Ipanema | Atlantic Forest | 2003 |
| VI | Floresta Nacional de Irati | Atlantic Forest | 2014 |
| VI | Floresta Nacional de Itaituba I e Itaituba II | Amazon | 2014 |
| VI | Floresta Nacional de Jacundá | Amazon | 2011 |
| VI | Floresta Nacional de Mapiá-Inauiní | Amazon | 2009 |
| VI | Floresta Nacional de Nísia Floresta | Atlantic Forest | 2012 |
| VI | Floresta Nacional de Pacotuba | Atlantic Forest | 2011 |
| VI | Floresta Nacional de Passa Quatro | Atlantic Forest | 2009 |
| VI | Floresta Nacional de Passo Fundo | Atlantic Forest | 2012 |
| VI | Floresta Nacional de Purus | Amazon | 2009 |
| VI | Floresta Nacional de Ritápolis | Atlantic Forest | 2005 |
| VI | Floresta Nacional de Saracá-Taquera | Amazon | 2002 |
| VI | Floresta Nacional de Tapajós | Amazon | 2005 |
| VI | Floresta Nacional de Tapirapé-Aquiri | Amazon | 2006 |
| VI | Floresta Nacional do Amana | Amazon | 2010 |
| VI | Floresta Nacional do Araripe-Apodi | Caatinga | 2005 |
| VI | Floresta Nacional do Crepori | Amazon | 2010 |
| VI | Floresta Nacional do Jamanxim | Amazon | 2011 |
| VI | Floresta Nacional do Jamari | Amazon | 2005 |
| VI | Floresta Nacional do Trairão | Amazon | 2011 |
| VI | **Extractive Reserves**  Reserva Extrativista Arapixi | Amazon | 2010 |
| VI | Reserva Extrativista Auatí-Paraná | Amazon | 2012 |
| VI | Reserva Extrativista Baixo Juruá | Amazon | 2009 |
| VI | Reserva Extrativista Cazumbá-Iracema | Amazon | 2008 |
| VI | Reserva Extrativista Chico Mendes | Amazon | 2008 |
| VI | Reserva Extrativista do Lago do Capanã Grande | Amazon | 2013 |
| VI | Reserva Extrativista do Rio Jutaí | Amazon | 2012 |
| VI | Reserva Extrativista Mandira | Marine-Coastal | 2011 |
| VI | Reserva Extrativista Marinha Caeté-Taperaçu | Marine-Coastal | 2013 |
| VI | Reserva Extrativista Médio Juruá | Amazon | 2012 |
| VI | Reserva Extrativista Rio Iriri | Amazon | 2011 |
| VI | Reserva Extrativista Rio Ouro Preto | Amazon | 2014 |
| VI | Reserva Extrativista Rio Unini | Amazon | 2014 |
| VI | Reserva Extrativista Rio Xingu | Amazon | 2012 |
| VI | Reserva Extrativista Riozinho do Anfrísio | Amazon | 2011 |
| VI | Reserva Extrativista Tapajós Arapiuns | Amazon | 2014 |
