# Supplementary material for: Environmental diagnoses and effective planning of Protected Areas in Brazil: Is there any connection?
Source: PLoS One. 2020 Dec 11;15(12):e0242687. doi: 10.1371/journal.pone.0242687 (PMC7732074; doi:10.1371/journal.pone.0242687)
Supplement: S2 Table — (DOCX) [file pone.0242687.s002.docx]

**S2 Table.** Criteria for classifying of data collection methods in the environmental diagnoses of management plans. In parentheses: percentage of PAs that used each method.

| *Methods* | *Criteria* |
| --- | --- |
| *Participatory workshops or interviews with residents or beneficiaries of the PA*  (15%) | *Considered when:*  ● Identify or map environmental elements of the PA;  ● Identify or map the occurrence and use of the PA’s natural resources;  ● Identify or map problems or threats to environmental order in the PA;  ● Presentation and validation of the results of other methods used for environmental diagnoses (*e.g.,* presentation of secondary data from the region or PA;  *Not considered when:*  ● Defined the methods or themes to be studied in the environmental diagnoses of the PA. |
| *Use of secondary data on the region of the PA*  (100%) | *Considered when:*  ● Used secondary data from the PA region (e.g., municipality, state, biome, neighboring PA etc.). The delimitation of the PA region is done by the team that prepares the management plan. This same team defines which secondary data obtained outside the PA can be extrapolated to the area, to subsidize PM. We only identify information about the use of data from the UC region in the management plan documents.  *Observation*:  When considering as secondary data any scientific or non-scientific publication, such as previous management plans, the creation process of the PA, technical reports of the managing body or of universities, scientific articles etc. |
| *Use of a few secondary data from the interior of the PA*  (75%) | *Considered when:*  ● There is a citation of secondary data from the interior of the PA by the authors, provided that the diagnosis has not been considered as “use of secondary data from a PA research program”;  ● The authors cite that existing secondary data were insufficient for planning, and a complementary field survey was carried out for the MP;  *Not considered when:*  ● There was a large quantity of secondary data from research programs or similar activities in the PA, according to the criteria of the respective method ¹;  ● Few secondary data referred to the management of a species, biological community, environment or the use of a natural resource of the PA¹. |
|  | *Observation*:  Any publication, scientific or not, such as previous management plans, the creation process of the PA, technical reports of the managing body or of universities, scientific articles, etc. were considered as secondary data. |
| *Use or performance of specific studies for the management of a species, community or environment of the PA*  (32%) | *Considered when:*  ● When specific studies have been carried out for the management or protection of a species, biological community or environment of the PA (including the use of natural resources in Sustainable Use PAs) specifically for the MP prior to its establishment;  ● Studies were conducted to survey or analyze threats to the environmental elements of the PA;  *Not considered when:*  ● When study results are not presented in the MP, even if there is a citation in the document that they were performed. |
| *Primary data from rapid surveys for the management plan*  (67%) | *Considered when:*  ● Rapid Ecological Assessments² were performed with or without adaptations, according to the citation of the authors of the MP;  ● Rapids surveys were performed without a specific method, generally with two or three field stages, and including two or more themes, even if studied separately;  ● Rapid surveys were performed according to the previous criteria, in order to support the zoning of the PA, even before the MP was drafted;  ● Surveys of more than three field stages were performed, given they have taken place in different locations of large-scale PAs (more than 2 million hectares). |
| *Primary data from long-term surveys for the management plan*  (2%) | *Considered when:*  Primary data surveys were performed with four or more field stages over 2.5 years or more. |
| *Use of secondary data from PA research programs*  (10%) | *Considered when:*  ● Secondary data from formal research programs of the PA on a variety of themes were used;  ● Secondary data from informal research programs in the interior of the PA on a variety of themes were used (when there is support or encouragement for the PA to perform research, or research of the PA by universities of the region), in accordance with the citation of the authors of the MP;  ● There is a citation by the authors of the MP that the PA has a large amount of secondary data from the interior, and these were considered sufficient for planning;  ● Secondary data from the interior of the PA from Environmental Impact Studies, or research programs of enterprises within or near the PA are used.  *Observation*:  Any scientific or non-scientific publication, such as previous management plans, the creation process of the PA, technical reports of the managing body or universities, scientific articles, etc., were considered as secondary data |
| ¹Criteria used to avoid replication of data in different methods. ²For more details [11]. | |
