# Supplementary material for: Environmental diagnoses and effective planning of Protected Areas in Brazil: Is there any connection?
Source: PLoS One. 2020 Dec 11;15(12):e0242687. doi: 10.1371/journal.pone.0242687 (PMC7732074; doi:10.1371/journal.pone.0242687)
Supplement: S3 Table — (DOCX) [file pone.0242687.s003.docx]

**S3 Table.** Criteria for classification of the analyses performed in the environmental diagnoses of the evaluated management plans.

| *Analyses* | *Definition* | *Criteria* |
| --- | --- | --- |
| *All analyses* | Evaluation or interpretation of the set of data obtained for the description of the PA for the generation of useful information to support planning and management. | ● Only the analyses presented in the environmental diagnoses of the PA were considered. Analyses carried out in the planning stage were not considered;  ● Exception made for sustainable use PAs, where the description of the uses of natural resources of the PA, usually presented in the socioeconomic diagnosis, were evaluated for the identification of threats to resources. |
| *Identification of threats* | Human actions that directly affect the environment of the PA (*e.g.,* hunting, unsustainable fishing, hydroelectric construction, deforestation, etc.) or indirectly through factors that contribute to increased direct threats (*e.g.,* lack of sustainable income alternatives, lack of enforcement, demand for wood, demand for electricity, etc.) Threats can also be natural phenomena, driven or not by human actions (*e.g.,* global warming and climate change), and may stem from social or economic demands on the PA [13]. | *Considered when:*  ● When direct or indirect threats, whether current or future, have been identified, regardless of the number of threats considered and the form of presentation in the document (systematically or scattered throughout the text of the environmental diagnosis);  ● Cited in the description of the direct uses of natural resources in Sustainable Use PAs that permitted use has caused or may cause damage to the resources exploited or to other environmental elements of the PA. |
| *Conservation targets* | Elements (species, biological communities, physical characteristics, habitats, ecosystems, ecological systems) that represent the entire biodiversity of the area and the actions developed for its maintenance tend to contribute to the conservation of the ecosystem of the PA as a whole. Conservation targets, together with threats to them, are the basis for the definition of management and conservation strategies of the PA [12,13]. | *Considered when:*  ● Emphasized the important biological or physical elements of the area, and treated by the authors as conservation targets according to the concept applied in the study. |
|  |  |  |
|  |  | *Observation:*  The environmental elements present in the declaration of significance were not considered when they were treated in a broad way and without prioritization among them, and/or not being in agreement with the concept used in the study |
| *Classification of PA environments* | Evaluation of different PA environments (*e.g.,* river basins, sampling regions, vegetation cover classes, habitat types, etc.) for their biological importance, state of conservation or vulnerability. This differentiation supports zoning, prioritization of areas of the PA, and spatially directs management actions and permitted uses [11]. | *Considered when:*  ● They present the classification of the PA environments for at least one of the three proposed categories, in a specialized way by maps or described with sufficient clarity of location within the PA. |
| *Classification of environments - biological importance* | Differentiation of PA environments by richness or diversity of species, occurrences of threatened or endemic species, habitat or ecosystem diversity, connectivity, landscape value or other factors that indicate the importance of the environment to PA biodiversity [11]. | *Considered when:*  ● Used one or more criteria quoted in the definition, in isolation or integrated, regardless of the number of themes used for classification. E*.g.,* diversity of bird species or richness and diversity of species of fauna and flora evaluated together;  ● Performed in conjunction with the state of conservation, as in the environmental categorization made in Rapid Ecological Assessments [11], which consider the criteria of these two analyses to present a single score for the studied areas. |
| *Classification of environments - vulnerability* | Differentiation of PA environments in terms of their fragility, usually based on elements of the physical environment, such as geological instability, vulnerability to erosion, flood risks, etc. They may also consider vegetative aspects, such as fire risk, fragility of vegetation or resilience of environments [19]. | *Considered when:*  ● Used one or more criteria quoted in the definition, in isolation or integrated, regardless of the number of themes used for classification. *E.g.,* susceptibility of soil to erosion, geology and declivity in in an integrated manner;  *Observation*:  Susceptibility to erosion was considered only when the degree of vulnerability was indicated for all soil types cited in the diagnosis. |
| *Classification of environments - state of conservation* | Differentiation of PA environments regarding their state of conservation, as assessed by the area covered and the habitat conditions of the PA (generally vegetation), or their vital functions (availability of water and nutrients), and the presence of indicator species [11,19]. | *Considered when:*  ● Used one or more criteria quoted in the definition, in isolation or integrated, regardless of the number of themes used for classification. *E.g.* mapping of the vegetation cover or use of the soil of the CA; presentation throughout the text characterizing the phytophysiognomies of percentages of deforestation or forms of land use, or specific texts detailing the quality of the PA environments based on flora and fauna;  ● Presentation of maps of the use of natural resources of Sustainable Use PAs, as they present the areas with greater and lesser exploitation of the resources of the PA;  ● Performed in conjunction with biological importance, as in the environmental categorization made in Rapid Ecological Assessments [11], which consider the criteria of these two analyses to present a unique relative score for the areas studied. |
| *Future management and conservation scenarios* | Assessment of the factors that may affect the PA in the future, whether they are threats (climate change, trends in public use, use of natural resources in the PA, economic and infrastructure pressures, etc.) or future opportunities (possibilities for environmental recovery, or positive changes in the socioeconomic environment in which the PA is inserted) to generate desired scenarios. They serve as support for the elaboration of strategies for recovery, reduction of fragilities or long-term conservation of environments [8,19]. | *Considered when:*  ● Provides predictions about the maintenance of the PA environment, based on threats or future opportunities, as long as it considers aspects of conservation (such as state of the environment, reduction of threats etc.) and not only aspects of PA management;  ● Presented and analysed in the environmental diagnosis of the PA. |
| *Integrated analysis of thematic data* | Interdisciplinary analysis of the data obtained for the description of the PA, resulting in elements to support planning [11]. | *Considered when:*  ● Presented clear results for management, such as the classification of the studied environments;  *Not considered when:*  ● Performed only a multidisciplinary description of the PA. |
